# Comparison of diffusion-weighted with T2-weighted imaging for detection of edema in acute myocardial infarction

**DOI:** 10.1186/1532-429X-15-90

**Published:** 2013-10-07

**Authors:** Anna Kociemba, Małgorzta Pyda, Katarzyna Katulska, Magdalena Łanocha, Andrzej Siniawski, Magdalena Janus, Stefan Grajek

**Affiliations:** 1 Magnetic Resonance Unit, I’st Department of Cardiology, University of Medical Sciences, Poznan, Poland; 2 Department of Radiology, University of Medical Sciences, Poznan, Poland

## Abstract

**Background:**

Recent studies, performed with the use of a commercially available diffusion weighted imaging (DWI) sequence, showed that they are sensitive to the increase of water content in the myocardium and may be used as an alternative to the standard T_2_-weighted sequences. The aim of this study was to compare two methods of myocardial edema imaging: DWI and T_2_-TIRM.

**Methods:**

The study included 91 acute and post STEMI patients. We applied a qualitative and quantitative image analysis. The qualitative analysis consisted of evaluation of the quality of blood suppression, presence of artifacts and occurrence of high signal (edema) areas. On the basis of edema detection in AMI and control (post STEMI) group, the sensitivity and specificity of TIRM and DWI were determined. Two contrast to noise ratios (CNR) were calculated: CNR_1_ - the contrast between edema and healthy myocardium and CNR_2_ - the contrast between edema and intraventricular blood pool. The area of edema was measured for both TIRM and DWI sequences and compared with the infarct size in LGE images.

**Results:**

Edema occurred more frequently in the DWI sequence. A major difference was observed in the inferior wall, where an edema-high signal was observed in 46% in T_2_-TIRM, whereas in the DWI sequence in 85%. An analysis of the image quality parameters showed that the use of DWI sequence allows complete blood signal suppression in the left ventricular cavity and reduces the occurrence of motion artifacts. However, it is connected with a higher incidence of magnetic susceptibility artifacts and image distortion. An analysis of the CNRs showed that CNR_1_ in T_2_-TIRM sequence depends on the infarct location and has the lowest value for the inferior wall. The area of edema measured on DWI images was significantly larger than in T_2_-TIRM.

**Conclusions:**

DWI is a new technique for edema detection in patients with acute myocardial infarction which may be recommended for the diagnosis of acute injuries, especially in patients with slow-flow artifacts in TIRM images.

## Background

Evaluation of AMI (acute myocardial infarction) with magnetic resonance imaging, allows detection of reversible and irreversible injury of the myocardium [1]. Cardiovascular Magnetic Resonance (CMR) in one examination provides an accurate assessment of myocardial function and morphology (edema, impaired microcirculation, haemorrhage, necrosis). Therefore, CMR has great potential as a diagnostic method in the acute phase of myocardial infarction, providing information on the degree and extent of reversible and irreversible myocardial damage [2].

T_2_ weighted sequences are commonly applied to detect myocardial edema [3-7]. Combined with Late Gadolinium Enhancement (LGE), T_2_ images allow the quantification myocardial salvage index and to distinguish between acute and chronic injure [6,8]. Turbo inversion recovery (TIRM) is most frequently used for edema evaluation [5-7,9]. Inversion pulses for fat and blood effectively suppress the signal from tissues with short relaxation time and enhance the signal from tissues with long T_1_ and T_2_ times (e.g. edematous myocardium). Despite all the advantages of T_2_ imaging, the above technique has some limitations. One of the main shortcomings of this breath-hold technique are motion artifacts, which blur the image in the case of non compliant patients. Partial blood suppression in the area of slow flow leads to a “slow flow artifact” resulting in high signal, which is often confused with sub-endocardial edema [10]. Dark-blood preparation pulse may also cause a significant signal loss and variations, which may result in an incorrect diagnosis. Although TIRM is widely used, other CMR sequences for edema imaging still requires optimalization and development.

Diffusion Weighted Imaging (DWI) is a well known technique in neuroradiology owing to its ability to detect ischemic regions in brain tissue [11]. Rapid development of magnetic resonance technology (high gradient amplitudes, parallel imaging, multichannel coils) and echo planar imaging has enabled the application of diffusion imaging in cardiac examinations [12-14]. Recently, it has been reported that DWI is sensitive to the increase in the water content in the myocardium in patients with acute myocardial infarction. Thus it may be an alternative to standard TIRM sequences [13]. Despite high sensitivity of diffusion images to motion artifacts (which is a major problem in the case of cardiac imaging), this sequence might have a wide range of applications in CMR, from characterization of myocardial muscle to understanding the process of remodelling after myocardial infarction [15].

The aim of this study was to assess myocardial edema with diffusion weighted imaging in patients with acute myocardial infarction and to compare it with commonly used (routinely in our lab) T_2_ turbo inversion recovery, black blood sequences - TIRM.

## Methods

### Patient population

We screened 125 consecutive patients with STEMI admitted to the Department of Cardiology, University of Medical Sciences in Poznan, between June and December 2011. STEMI was defined as the presence of continuous chest pain for at least 30 min, ST-segment elevation in 2 or more contiguous ECG leads (≥ 1 mm for the arm leads and ≥ 2 mm for the precordial leads), elevated markers of myocardial necrosis (troponin I–TnI or creatinine kinase MB– CK-MB), the presence of coronary artery occlusion or significant stenosis with visible thrombus on initial coronary angiogram. All patients were treated with primary PCI in accordance with current STEMI guidelines. We excluded patients with STEMI and previous myocardial infarction, patients in critical clinical condition (i.e. with balloon counter pulsation or mechanical ventilation), patients with acute or chronic renal failure (contraindication to gadolinium contrast), or with other contraindications to CMR (i.e. temporary or permanent pacemakers, severe form of claustrophobia or metallic foreign objects in the body). The final group numbered 91 patients.

•control group - patients who were subjected to magnetic resonance imaging at least 6 months after myocardial infarction, n = 20;

•AMI group - patients who were subjected to magnetic resonance imaging not later than 7 days after myocardial infarction, n = 71.

All patients expressed a written consent to participate in the examination and a protocol was approved by the Bioethical Committee of the University of Medical Sciences in Poznan.

### Cardiovascular magnetic resonance

CMR was performed on 1.5 Tesla scanner (Magnetom Avanto, Siemens) with the use of a six-channel phased-array body coil combined with a six-channel spine matrix coil. The study protocol consisted of the following sequences: scout images in coronal, sagittal and transverse planes performed to locate the anatomical axes of the heart. Steady State Free Precession (SSFP) cine imaging was performed for wall motion and left ventricular function assessment in 2-, 3-, 4-chamber and short axis planes. T_2_ triple inversion recovery (TIRM) and DWI sequences were applied before contrast administration for detection of myocardial edema. Both sequences were acquired at the same location. Details of the compared sequences are shown in Table 1. The first pass imaging was acquired with the use of T1-weighed single shot, gradient echo planar imaging (GRE-EPI), at rest, after administration of 0.1 mmol/kg of gadolinium contrast agent. For LGE imaging we used segmented inversion recovery turbo flash sequence: TR-782 ms, TE-3.21 ms, slice thickness 10 mm, matrix 380 × 310.

**Table 1:** Sequence parameters for TIRM and DWI

	**TIRM**	**DWI**
**FoV read**	380 mm	380 mm
**FoV phase**	81.30%	81.30%
**Slice thickness**	10 mm	10 mm
**TR**	2xRR	3-4 s
**TE**	101 ms	78 ms
**Resolution**	256 × 208	192 × 188
**PAT mode**	GRAPPA (2)	GRAPPA (2)
**Averages**	1	4
**Bandwidth**	253 Hz/Px	1736 Hz/Px
**Echo spacing**	6.74 ms	0.69 ms
**ECG Trigger**	+	+
Navigator*	-	+
**b-value**	-	50;100;200 s/mm2

*FoV* – field of view, *TR*- repetition time,*TE* – echo time, *PAT* – parallel acquisition technique, *PACE - Motion under Control with Prospective Acquisition Correction.

### Image analysis

All CMR images were analysed on a dedicated workstation (Siemens, Medical Solution) by two separate observers. Analysis of left ventricular ejection fraction was performed using Argus software and volumetric method.

Initially, both AMI and control group patients were screened for the presence or absence of edema. To avoid interpretation bias, TIRM and DWI images were presented to the observers separately in random order. Moreover, in order to achieve blinding, the reviewer did not know patients’ clinical and angiographic data, or the assessment provided by the other observer. Sensitivity and specificity of TIRM and DWI were determined on the basis of the edema detected in AMI and control groups. Each patient in the AMI group was assumed to show an increased signal in the infarct area, while in the control group an increased signal should not be present.

Next, a detailed qualitative and quantitative image analysis was performed in AMI group (n = 71). For this purpose matching short axis slices were compared across SSFP, TIRM, DWI and LGE images.

The qualitative analysis consisted of evaluation of the quality of blood suppression, presence of motion and other artifacts (susceptibility, distortion, voiding) and the occurrence of high signal (edema) areas.

For the quantitative analysis, endo- and epicardial borders were manually outlined and signal intensity with standard deviation for four areas was measured: healthy muscle, the area of edema, left ventricular cavity and the background. Normal myocardium was defined as segments with normal regional wall thickening in retro images and the absence of contrast enhancement in LGE images. For TIRM and LGE images, a high signal of edema/infarction was defined as a myocardial signal intensity exceeding the mean value + 2 × standard deviation (SD) of noninfarcted, normal myocardium [6,16]. In order to asses area of edema, the region of interested (ROI) was selected within segments with visually normal signal intensity, normal regional wall thickening in retro images, and the absence of contrast enhancement in LGE images. The mean signal intensity and SD were measured. The image window was then adjusted to mean value + 2 × SD threshold and hyperintense areas were manually traced. Small, focal hyperintense (<5 pixels) areas were not considered as a part of edema. To standardize the definition of high edema signal in diffusion images, the edema was first assessed visually. Window settings were adjusted individually to the observer preferences and the area of edema and mean signal intensity for healthy myocardium was measured. The measurements of the area of edema were then repeated after thresholding the window settings at 1,2,3,4 and 5 SD above the intensity of the mean signal of a normal, non-infarcted myocardium. The highest agreement with visually assessed area was achieved for 2 SD and this threshold was used for DWI in this paper.

For quantitative evaluation of DWI and TIRM images in AMI group we used the method described by Deux [13]. Two contrast-to-noise ratios were calculated: CNR_1_ - the contrast between edema and healthy myocardium and CNR_2_ - the contrast between edema and intraventricular blood pool. The area of edema was measured for both TIRM and DWI sequences and compared with the infarct size in LGE images in the same slice position. Results are presented as a percentage of the left ventricle slice area.

We also examined the correlation between the area of edema in TIRM and DWI sequences with the area of fibrosis measured in LGE images. In order to visualize the area of edema that was smaller than the area of fibrosis, we applied the line of identity which denoted edema equal to fibrosis. Points located below the line of identity represent cases in which the designated area of edema was smaller than the area of fibrosis.

**Figure 1:**
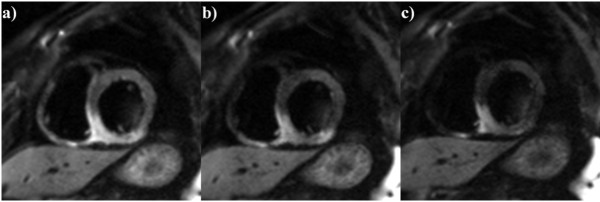
**Short axis view of the inferior wall edema.** CMR was performed 2 days after myocardial infarction. Diffusion weighted imaging with **a)** b = 50 mm/s^2^, **b)** b = 100 mm/s^2^, **c)** b = 200 mm/s^2^ acquired in a short axis plane.

DW images with b-value 50 s/mm2 were chosen for all the calculations as they showed the highest signal intensity for edema and healthy myocardium (Figure 1).

Because of the differences in the sequence parameters between DWI and TIRM the measurements of the signal intensity in TIRM was multiplied by a factor of 0.93 [13,17].

### Statistical analysis

The consensus of the observers’ assessments and the mean measurement value estimated by them were analyzed. Values of continuous variables are expressed as a mean (±SD) and a median (range). Normality was confirmed or excluded using the Shapiro-Wilk test. Continuous variables were compared using Mann Whitney or Wilkoxon test and noncontiguous data using chi-square test. Statistical significance was defined as p < 0.05.

## Results

The study included 91 patients, 71 in AMI and 20 in the control group. Groups did not differ regarding basic clinical data (Table 2).

**Table 2:** Comparison of basic clinical data in the study and control group

**Parameter**	**AMI group**	**Control group**	***p***
**n**	71	20	
**Age (years)**	60.2 ± 11.9 59.5 (35.0-84.0)	60.1 ± 7.9 60.5 (49.0-76.0)	ns
**Men *n *(%)**	53 (75%)	17 (85%)	ns
**Women *n *(%)**	18 (25%)	3 (15%)	ns
**EF (%)**	49.9 ± 11.6 51.1 (19–72)	45.9 ± 17.2 55 (14–69)	ns
**SV (ml)**	78.1 ± 21.7 75.6 (39.9-151.0)	92.4 ± 23.0 92 (38–128)	ns
**Infarct location:**			
**LAD**	23 (32%)	10 (50%)	ns
**RCA**	41 (58%)	6 (30%)	
**LCX**	7 (10%)	4 (20%)	
**TIMI pre PCI:**			
**TIMI - 0**	53 (75%)	13 (65%)	ns
**TIMI - 1**	8 (11%)	3 (15%)	
**TIMI - 2**	7 (10%)	3 (15%)	
**TIMI - 3**	3 (4%)	1 (5%)	
**TIMI post PCI**			
**TIMI - 0**	1 (1%)	0 (0%)	ns
**TIMI - 1**	2 (3%)	1 (5%)	
**TIMI - 2**	16 (22%)	3 (15%)	
**TIMI - 3**	52 (74%)	16 (80%)	
**PCI - CMR time (days)**	2.5 ± 1.5 2 (1–7)	293 ± 42 298 (235–354)	<0.001
**Time to pPCI (hours)**	5.1 ± 3.7 5 (1–12)	4.8 ± 4.1 5 (2–12)	ns
**Value: mean ± SD median (min-max); *n *(%)**			

The intraobserver reproducibility for qualitative evaluation was 95%. The analysis showed that DWI has a higher sensitivity (83.1%) than TIRM sequence (60.6%) with the same specificity of 90%. The negative predictive value was found to be higher in the DWI sequence (60.0%) than in TIRM images (39.1%). Both sequences had the positive predictive value of approximately 96%.

The analysis of the quality parameters of TIRM and DWI of the AMI group showed a significant difference in signal suppression of the left ventricle blood signal. In TIRM sequence, suppression was accomplished in 50 out of 71 patients, while in DWI in all cases. The occurrence of motion artifacts was significantly higher in the TIRM images 24%, relative to DWI 3%. There was also a significant difference in the frequency of susceptibility artifacts (in DWI occurred almost 12 times more often than in the TIRM sequence). Most significant, high signal of myocardial edema was detected in 43 (58%) of the patients in TIRM and 59 (80%) in DWI (Table 3). Detectability of the high signal depending on the infarct location was also analyzed. This revealed that a DWI sequence detects edema in the inferior wall much more frequently than TIRM sequence. The sensitivity of TIRM sequence was only 46% in RCA territory as compared to 85% for DWI (Table 4).

**Table 3:** Results of a qualitative analysis of TIRM and DWI images in AMI patients

	**TIRM**	**DWI**	**p**
**Blood suppression**	50 (70%)	71 (100%)	<0.0001
**Motion artifacts**	17 (24%)	2 (3%)	0.0007
**Other artifacts**	2 (3%)	23 (32%)	<0.0001
**High signal presence**	43 (61%)	59 (83%)	0.0238
**n(%)**			

**Table 4:** Detection of high signal areas in TIRM and DWI images depending on infarction location

	**TIRM**	**DWI**	**p**
**LAD**	18 (78%)	18 (78%)	ns
**LCX**	6 (86%)	6 (86%)	ns
**RCA**	19 (46%)	35 (85%)	0.0002
**n(%)**			

The overall contrast ratio between the area of edema and healthy myocardium (CNR_1_) and the contrast ratio between the area of edema and blood inside the left ventricle (CNR_2_) did not differ between DWI and TIRM sequences (Table 5). The analysis of CNR_1_ and CNR_2_ for both sequences according to infarct location showed a significant relation in contrast ratio between the area of edema and the healthy muscle (CNR_1_) in TIRM sequence (p = 0.0442). The highest value of CNR_1_ occurred in the area supplied by the left descending coronary artery (CNR_1_ = 21.4 ± 7.1), while the lowest in the area supplied by the right coronary artery (CNR_1_ = 15.4 ± 6.3) (Table 6).

**Table 5:** Contrast to noise ratios (CNR) in AMI patients

	**TIRM**	**DWI**	**p**
**CNR**_**1**_	18.2 ± 7.4 17.3 (5.3-39.4)	21.4 ± 11.5 19.7 (2.7-58.9)	ns
**CNR**_**2**_	29.0 ± 14.9 26.4 (4.5-72.8)	31.2 ± 20.0 26.7 (6.0-117.2)	ns
**Value: mean ± SD median (min-max)**			

**Table 6:** **Comparison of the CNR**_**1 **_**and CNR**_**2 **_**for DWI and TIRM sequences depending on infarction location**

	**LAD**	**LCX**	**RCA**	**p**
**CNR**_**1 **_**TIRM**	21.4 ± 7.1 19.7 (12.3-39.4)	18.0 ± 8.8 18.2 (7.8-27.6)	15.4 ± 6.3 14.9 (5.3-32.7)	0.0442
**CNR**_**2 **_**TIRM**	30.4 ± 14.3 30.6 (6.0-61.7)	33.5 ± 20.6 25.6 (18.1-71.8)	26.3 ± 13.7 25.4 (4.5-72.8)	ns
**CNR**_**1 **_**DWI**	19.7 ± 5.9 19.3 (11.1-33.3)	12.7 ± 7.8 12.4 (2.7-25.7)	23.8 ± 13.3 21.2 (3.8-58.9)	ns
**CNR**_**2 **_**DWI**	31.4 ± 11.8 27.7 (19.4-65.7)	21.7 ± 20.4 12.8 (8.8-62.3)	32.7 ± 23.1 27.4 (6.0-117.2)	ns
**Value: mean ± SD median (min-max)**				

**Figure 2:**
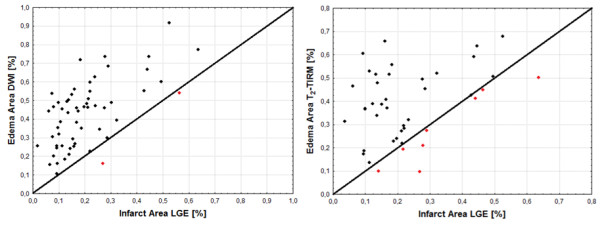
Scatterplots for relationships between infarct size and edema derived from DWI and TIRM.

The average area of edema measured in DWI images 48.1 ± 1.8% was significantly larger than in TIRM images 37.1 ± 1.7%, p = 0.0004. In both, TIRM and DWI, the area of edema was significantly larger than the area of fibrosis in LGE sequences 22.9 ± 1.4%. A stronger correlation between the areas of edema and fibrosis was observed for DWI images (r = 0.61, p <0.001). For TIRM, the values were r = 0.39, p = 0.01. In TIRM images, in 8 patients edema was smaller than fibrosis in LGE images. In DWI this difference was observed in two patients only (Figure 2).

## Discussion

Our study indicates that diffusion weighted imaging detects areas of increased myocardial signal in patients with acute myocardial infarction with high sensitivity and specificity (83.1% and 90.0% respectively). DWI may also be used to differentiate chronic from acute injury. Other authors have reported the presence of edema in DWI in all patients in the acute phase of myocardial infarction [12,13]. However, these studies involved only small groups of patients. Our study is the first to report the sensitivity and specificity in the detection of edema with DWI sequence in a larger group of patients and has shown a lower sensitivity and specificity of T_2_-TIRM than previously reported (60, 6%, 90%, respectively). Abel-Aty et al. reported 91-94% and 92-100% (sensitivity and specificity, respectively), differentiating between the acute and chronic myocardial infarction with T_2_-TIRM and LGE [6]. Another study reported that T_2_-TIRM allows detection of acute myocardial infarction with 100% sensitivity and specificity [5]. Payne et al. compared the diagnostic accuracy of T_2_-TIRM and ACUT2E and found that only in 61% of cases T_2_-TIRM allowed proper identification of infarct-related artery. The sensitivity was 96% and specificity of detection of the anterior wall edema and not more than 35% [3]. However, because that study covered both STEMI and NSTEMI patients, the results cannot be straightforwardly compared. Differences in sensitivity and specificity may have been caused by different sequence parameters used (slice thickness, signal averages etc.) in these studies. The use of thicker layers (e. g. 15 mm) leads to an increased signal to noise ratio but it also increases the incidence of slow flow artifacts. The application of thinner layers (around 8–10 mm) reduces the partial volume effect and allows to detect small edema areas. Also selection of an appropriate TE has a considerable impact on the detection of edema with T2 imaging. Longer TE provides better contrast but decreases SNR [18]. In this study we used TIRM with TE of 101 ms and slice thickness of 10 mm as these parameters are routinely used in our department. However, alternatives are also possible.

The sensitivity of DWI sequence in our study did not depend on infarct location. Contrary to the DWI, the TIRM sensitivity in RCA territory was significantly lower than in other areas (Table 4). Also CNR_1_ in TIRM depended on infarct location (Table 6). Our results support that TIRM signal intensity depends on the distance from the coil, especially when a surface coil is used. A close location of surface coil to certain segments of myocardium (e.g. anterior wall) may lead to misinterpretation of the signal as pathological [19], while edematous areas in the inferior wall may be diagnosed as normal. The solution to this problem is the use of body coil. This provides better signal uniformity but is associated with a decrease in signal-to-noise ratio and prevents the use of parallel imaging techniques which extends the scan time.

**Figure 3:**
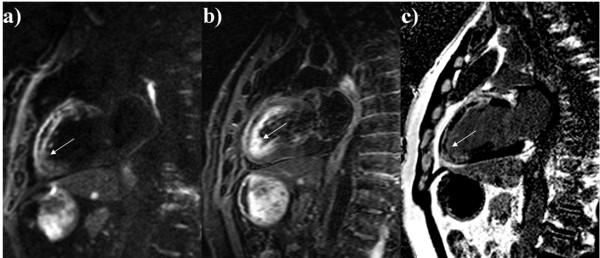
**CMR performed 3 days after myocardial infarction.** Diffusion weighted imaging in 2-chamber view **a)**; slow flow artifact in TIRM image in the same slice position **b)**; delayed-enhanced image, acquired at the same level, showed a high signal area located in segments supplied by the left anterior descending artery **c)**; late gadolinium enhancement image acquired at the same level. **c)** late gadolinium enhancement image acquired at the same level.

In our study, the DWI sequence nullified the blood pool signal in all cases, whereas TIRM images in 70% (Figure 3). In TIRM images the slow flowing blood caused by akinesis may not be adequately suppressed and therefore it appears as a high signal. This phenomenon is often confused with subendocardial edema [20].

We also found that the mean size of edema-high signal measured in DWI was significantly larger than in TIRM images. This difference may be due to the fact that the signal in the DWI sequence is influenced by two factors: relaxation time of water in the area of edema and the cardiac perfusion. Because the DWI is a T_2_-weighted sequence, the high intensity of the edema signal is caused by a long relaxation time of water in cardiomyocytes. This phenomenon may be sometimes reduced by a selection of an appropriate TE (short) and b value (high). However, in this study b = 50 mm2/s was used, therefore the role of T_2_ relaxation in DWI signal intensity is significant. Also high signal intensity in the infarction area reflects the perfusion disorder in myocardium. Diffusion-weighted imaging performed in a well-vascularized tissue results in attenuation of signal observed at low b values (e.g. 0–100 s/mm2), as the capillary blood flow analyzed in a voxel scale, may be mistaken for a type of diffusion. Since all AMI patients had abnormal microvascular perfusion due to acute myocardial infarction, we cannot exclude that this effect influenced the detection of the increased signal. Further, it cannot be excluded that DWIs provided some false positive results due to the bulk motion artifact. Diffusion imaging using EPI is sensitive to several artifacts, of which geometric distortions are most frequent. Those distortions, induced by magnetic susceptibility differences across the imaging tissues and bulk motions appearing during the application of the diffusion gradients, may lead to inhomogeneous signal attenuation artifacts (signal dropouts) in DWI [21]. These artifacts may result in a greater decrease in signal intensity in well-contracting myocardial segments. The Apparent Diffusion Coefficient (ADC) calculation may provide additional, quantitative information on both, the cytotoxic and vasogenic myocardial edema. The ADC map can be calculated by acquiring two or more images with different b-values. The contrast in the ADC map depends on the spatially distributed diffusion coefficient of the acquired tissues and does not contain T_1_ and T_2_ values as is the case in single diffusion weighted images. However, since the impact of all factors on myocardial signal intensity in patients with acute myocardial infarction has not yet been fully examined, the analysis of ADC maps is beyond the scope of this paper.

We have also shown that the area of edema measured in T_2_-TIRM and DWI correlates with the area of fibrosis in LGE images. The correlation is much stronger for DWI sequence (r = 0.67 vs r = 0.39 in T_2_-TIRM). In addition, the analysis showed that in T_2_-TIRM in 8 patients, the area of edema was smaller than that of fibrosis. In DWI this difference occurred only in two patients. Since the area of edema should always be greater than, or equal to, the area of fibrosis, it must be recognized that the application of T_2_-TIRM will more frequently underestimate the area of edema.

### Limitations

This study has three main limitations:

In our study the minimum pPCI to CMR time in the control group was 235 days. Previous studies showed that in some patients, small edema lesions might still be present 6 or even 12 months after AMI [22-24]. Consequently, the presence of edema according to the control group, cannot be completely excluded. Reported persistence of myocardial edema in CMR varies. This can be explained by different clinical courses after AMI or by misinterpretation of the image due to artifacts.

TIRM sequence was used as a reference method for DWI although recent studies have shown that bright blood T_2_ imaging has a higher diagnostic accuracy than application of TIRM in AMI [25-27]. Also, direct measurements of relaxation times by T1 or T2 mapping [28,29] are a promising methods of edema estimation. Therefore, an ideal approach would be to compare DWI, TIRM and T2/T1 maps, and T_2_-prepared SSFP. However, since T_2_-TIRM continues to be widely used in most centers and has acceptable reproducibility [30], the method used in this study seems to be relevant.

This study evaluates two different methods for edema imaging. In such a study full blindness is cumbersome to achieve due to the specific characteristics of the images which are practically impossible to conceal. This has a negative effect on the qualitative analysis while the quantitative analysis, based on objective parameters, is less affected.

## Conclusions

Diffusion weighted imaging is a new technique for edema detection in patients with acute myocardial infarction and has a higher sensitivity in the detection of myocardial edema than T_2_-TIRM. The sensitivity of the DWI sequence, in contrast to the T_2_-TIRM, does not depend on the infarct location, provides total suppression of the blood signal and reduces the occurrence of motion artifacts. DWI may be recommended for the diagnosis of acute injuries, especially in patients with “slow-flow” artifacts in TIRM images. The results of our study require further investigation, especially in light of a relatively few papers on diffusion imaging of the heart. It should also be remembered that the diffusion weighted imaging is more challenging and demanding for the technical staff. It is more time consuming and requires manual adjustment of the acquisition window, depending on the patient heart rate. However, these obstacles can be eliminated by introducing a diffusion sequence dedicated to cardiac imaging, thus allowing automatic adjustment of the acquisition window.

## Abbreviations

ACU2E: Acquisition for cardiac unified T2 edema; ADC: Apparent diffusion coefficient; AMI: Acute myocardial infarction; CMR: Cardiovascular magnetic resonance; CNR: Contrast to noise ratio; DWI: Diffusion weighted imaging; EPI: Echo planar imaging; FoV: Field of view; LAD: Left coronary artery; LCX: Left Circumflex Artery; LGE: Late gadolinium enhancement; LV: Left ventricle; NSTEMI: Non ST elevation myocardial infarction; PACE: Motion under control with prospective acquisition correction; PAT: Parallel acquisition technique; pPCI: Primary percutaneous coronary intervention; RCA: Right coronary artery; SD: Standard deviation; SNR: Signal to noise ratio; SSFP: Steady state free precession; STEMI: ST elevation myocardial infarction; T2-TIRM: T2 turbo inversion recovery with magnitude display; TE: Echo time; TR: Repetition time.

## Competing interests

The authors declare that they have no competing interests.

## Authors’ contributions

AK was involved in developing the sequence, collecting and analysing the data, drafting and submitting the manuscript. MP was involved in designing the study, revising and submitting the manuscript. KK was involved in designing the study and developing the sequence. MŁ, AS MJ were involved in evaluating the patients studies. SG was involved in overseeing the study. All authors have read and approved the final manuscript.

## References

[B1] Abdel-Aty H Tillmanns C The use of cardiovascular magnetic resonance in acute myocardial infarction Curr Cardiol Rep 2010 12 1 76 81 10.1007/s11886-009-0076-y 20425187

[B2] Friedrich MG Tissue characterization of acute myocardial infarction and myocarditis by cardiac magnetic resonance JACC Cardiovasc Imaging 2008 1 5 652 62 10.1016/j.jcmg.2008.07.011 19356496

[B3] Wassmuth R Prothmann M Utz W Variability and homogeneity of cardiovascular magnetic resonance myocardial T2-mapping in volunteers compared to patients with edema J Cardiovasc Magn Reson 2013 15 27 10.1186/1532-429X-15-27 23537111 PMC3627620

[B4] Joey FA U Henrik E David E Cardiovascular magnetic resonance of the myocardium at risk in acute reperfused myocardial infarction: comparison of T2-weighted imaging versus the circumferential endocardial extent of late gadolinium enhancement with transmural projection J Cardiovasc Magn Reson 2010 12 1 18 10.1186/1532-429X-12-18 20350309 PMC2855565

[B5] Friedrich MG Abdel-Aty H Taylor A The salvaged area at risk in reperfused acute myocardial infarction as visualized by cardiovascular magnetic resonance J Am Coll Cardiol 2008 51 16 1581 7 10.1016/j.jacc.2008.01.019 18420102

[B6] Abdel-Aty H Zagrosek A Schulz-Menger J Delayed enhancement and T2-weighted cardiovascular magnetic resonance imaging differentiate acute from chronic myocardial infarction Circulation 2004 109 20 2411 6 10.1161/01.CIR.0000127428.10985.C6 15123531

[B7] H-Ici DO Ridgway JP Kuehne T Cardiovascular magnetic resonance of myocardial edema using a short inversion time inversion recovery (TIRM) black-blood technique: diagnostic accuracy of visual and semi-quantitative assessment J Cardiovasc Magn Reson 2012 14 1 22 10.1186/1532-429X-14-22 22455461 PMC3350411

[B8] Aletras AH Tilak GS Natanzon A Retrospective determination of the area at risk for reperfused acute myocardial infarction with T2-weighted cardiac magnetic resonance imaging: histopathological and displacement encoding with stimulated echoes (DENSE) functional validations Circulation 2006 113 15 1865 70 10.1161/CIRCULATIONAHA.105.576025 16606793

[B9] Eitel I Friedrich MG T2-weighted cardiovascular magnetic resonance in acute cardiac disease J Cardiovasc Magn Reson 2011 13 1 13 10.1186/1532-429X-13-13 21332972 PMC3060149

[B10] Abdel-Aty H Simonetti O Friedrich MG T2-weighted cardiovascular magnetic resonance imaging J Magn Reson Imaging 2007 26 3 452 9 10.1002/jmri.21028 17729358

[B11] Lövblad KO Laubach HJ Bairda E Clinical experience with diffusion-weighted MR in patients with acute stroke AJNR Am J Neuroradiol 1998 19 6 1061 6 9672012 PMC8338657

[B12] Laissy J Gaxotte V Pasi N Cardiac diffusion-weighted MR imaging in recent, subacute and chronic myocardial infarction: a pilot study J Magn Reson Imaging 2011 13 Suppl 1 O46 10.1002/jmri.24125 23564654

[B13] Deux J-F Maatouk M Vignaud A Diffusion-weighted echo planar imaging in patients with recent myocardial infarction Eur Radiol 2011 21 1 46 53 10.1007/s00330-010-1912-6 20680287

[B14] Nensa F Mahabadi AA Erbel R Myocardial edema during acute myocardial infarction visualized by diffusion-weighted MRI Herz 2013 38 5 509 510 10.1007/s00059-012-3705-y 23263243

[B15] Wu M-T Tseng W-YI Su M-YM Diffusion tensor magnetic resonance imaging mapping the fiber architecture remodeling in human myocardium after infarction: correlation with viability and wall motion Circulation 2006 114 10 1036 45 10.1161/CIRCULATIONAHA.105.545863 16940196

[B16] Wright J Adriaenssens T Dymarkowski S Quantification of myocardial area at risk with T2-weighted CMR: comparison with contrast-enhanced CMR and coronary angiography JACC Cardiovasc Imaging 2009 2 7 825 31 10.1016/j.jcmg.2009.02.011 19608131

[B17] Haacke M Brown W Thompson M Chapter 15: Signal Contrast and Noise Magnetic Resonance Imaging Physical Principles and Sequence Design 1999 New York: John Wiley and Sons 347

[B18] Lønborg J Vejlstrup N Mathiasen AB Myocardial area at risk and salvage measured by T2-weighted cardiovascular magnetic resonance: reproducibility and comparison of two T2-weighted protocols J Cardiovasc Magn Reson 2011 13 1 50 10.1186/1532-429X-13-50 21917186 PMC3184621

[B19] Abdel-Aty H Schulz-Menger J Cardiovascular magnetic resonance T2-weighted imaging of myocardial edema in acute myocardial infarction Recent Pat Cardiovasc Drug Discov 2007 2 1 63 8 10.2174/157489007779606167 18221104

[B20] Arai AE Using magnetic resonance imaging to characterize recent myocardial injury: utility in acute coronary syndrome and other clinical scenarios Circulation 2008 118 8 795 6 10.1161/CIRCULATIONAHA.108.797373 18711021 PMC2764357

[B21] Benner T van der Kouwe AJW Sorensena G Diffusion imaging with prospective motion correction and reacquisition Magn Reson Med 2011 66 1 154 67 10.1002/mrm.22837 21695721 PMC3121006

[B22] Dall’Armellina E Karia N Lindsay AC Dynamic changes of edema and late gadolinium enhancement after acute myocardial infarction and their relationship to functional recovery and salvage index Circ Cardiovasc Imaging 2011 4 3 228 3 10.1161/CIRCIMAGING.111.963421 21447711 PMC3098134

[B23] Nilsson JC Nielsen G Groenning BA Sustained postinfarction myocardial oedema in humans visualised by magnetic resonance imaging Heart 2001 85 639 642 10.1136/heart.85.6.639 11359743 PMC1729755

[B24] Ripa RS Nilsson JC Wang Y Short- and long-term changes in myocardial function, morphology, edema, and infarct mass after ST-segment elevation myocardial infarction evaluated by serial magnetic resonance imaging Am Heart J 2007 154 929 936 10.1016/j.ahj.2007.06.038 17967600

[B25] Payne AR Casey M McClure J Bright-blood T2-weighted MRI has higher diagnostic accuracy than dark-blood short tau inversion recovery MRI for detection of acute myocardial infarction and for assessment of the ischemic area at risk and myocardial salvage Circ Cardiovasc Imaging 2011 4 3 210 9 10.1161/CIRCIMAGING.110.960450 21427362

[B26] Berry C Kellman P Mancini C Magnetic resonance imaging delineates the ischemic area at risk and myocardial salvage in patients with acute myocardial infarction Circ Cardiovasc Imaging 2010 3 5 527 35 10.1161/CIRCIMAGING.109.900761 20631034 PMC2966468

[B27] Payne AR Berry C Kellman P Bright-blood T(2)-weighted MRI has high diagnostic accuracy for myocardial hemorrhage in myocardial infarction: a preclinical validation study in swine Circ Cardiovasc Imaging 2011 4 6 738 45 10.1161/CIRCIMAGING.111.965095 21930836 PMC4158314

[B28] Verhaert D Thavendiranathan P Giri S Direct T2 quantification of myocardial edema in acute ischemic injury JACC Cardiovasc Imaging 2011 4 3 269 78 10.1016/j.jcmg.2010.09.023 21414575 PMC4282779

[B29] Ugander M Bagi PS Oki AJ Myocardial edema as detected by pre-contrast T1 and T2 CMR delineates area at risk associated with acute myocardial infarction JACC Cardiovasc Imaging 2012 5 6 596 603 10.1016/j.jcmg.2012.01.016 22698528 PMC3769169

[B30] Desch S Engelhardt H Meissner J Reliability of myocardial salvage assessment by cardiac magnetic resonance imaging in acute reperfused myocardial infarction Int J Cardiovasc Imaging 2012 28 2 263 72 10.1007/s10554-011-9802-9 21279689

